# A human monoclonal autoantibody to breast cancer identifies the PDZ domain containing protein GIPC1 as a novel breast cancer-associated antigen

**DOI:** 10.1186/1471-2407-8-248

**Published:** 2008-08-24

**Authors:** Sergei Rudchenko, Matthew Scanlan, Gavreel Kalantarov, Victoria Yavelsky, Chen Levy, Alison Estabrook, Lloyd Old, Gerald L Chan, Leslie Lobel, Ilya Trakht

**Affiliations:** 1 Hospital for Special Surgery, 535 East 70th Street, New York NY 10021, USA; 2 Ludwig Institute for Cancer Research, New York Branch at Memorial Sloan-Kettering Cancer Center, 1275 York Avenue, New York, NY 10021, USA; 3 College of Physicians and Surgeons, Columbia University, 630 W. 168 St., New York, NY 10032, USA; 4 Department of Virology, Faculty of Health Sciences, Ben Gurion University of the Negev, Beer Sheva 84105, Israel; 5 The Morningside Foundation, 1188 Centre Street, Newton Centre, MA 02459, USA

## Abstract

**Background:**

We have been studying the native autoimmune response to cancer through the isolation of human monoclonal antibodies that are cancer specific from cancer patients. To facilitate this work we previously developed a fusion partner cell line for human lymphocytes, MFP-2, that fuses efficiently with both human lymph node lymphocytes and peripheral blood lymphocytes. Using this unique trioma fusion partner cell line we isolated a panel of autologous human monoclonal antibodies, from both peripheral blood and lymph node lymphocytes, which are representative of the native repertoire of anti-cancer specific antibodies from breast cancer patients.

**Methods:**

The current study employs immunocytochemistry, immunohistochemistry, Western blot analysis as well as Northern blots, Scatchard binding studies and finally SEREX analysis for target antigen identification.

**Results:**

By application of an expression cloning technique known as SEREX, we determined that the target antigen for two monoclonal antibodies, 27.B1 and 27.F7, derived from lymph node B-cells of a breast cancer patient, is the PDZ domain-containing protein known as GIPC1. This protein is highly expressed not only in cultured human breast cancer cells, but also in primary and metastatic tumor tissues and its overexpression appears to be cancer cell specific. Confocal microscopy revealed cell membrane and cytoplasmic localization of the target protein, which is consistent with previous studies of this protein.

**Conclusion:**

We have determined that GIPC1 is a novel breast cancer-associated immunogenic antigen that is overexpressed in breast cancer. Its role, however, in the initiation and/or progression of breast cancer remains unclear and needs further clarification.

## Background

In patients with cancer, the body mounts an immune response following the onset of malignant disease since the new cells are recognized as non-self. It is composed of both immune cells that mediate innate, non-specific immunity, and adaptive, antigen-specific immunity [1-3]. Tumor cell proteins can elicit an immune response for various reasons; aberrant gene expression (e.g. cancer-testis antigens) [4-10], overexpression (neu/Her2) [11,12], aberrant processing (mucin) [13,14] and mutation events (p53) [11,15]. Although it is evident that a natural humoral response to cancer exists, tumor-associated antigens (TAAs) are generally notoriously bad immunogens. This is likely due to systemic tolerance to the autoantigens and, as a result, the natural humoral immune response against tumor antigens fails to reach high antibody titers and is not effective [16].

During the last decade, the search for TAAs that can be targeted by the immune system, and as such are "immunovisible", has been the focus of much research in cancer immunology. In addition, the isolation and production of fully human monoclonal antibodies (fhMAb) to such antigens has also made significant advances over the past few years [16-18]. The potential utility of these antibodies to identify TAAs, to discriminate between neoplastic and normal tissues and potentially act as anti-cancer therapeutics has been the impetus for this work.

To identify tumor-associated antigens, one of the more fruitful approaches has been to employ naturally occurring anti-cancer antibodies that arise in cancer patients. To this end, serological expression technology (SEREX) has facilitated the identification of novel TAAs by screening patients' whole sera on cDNA expression libraries that were prepared from autologous tumors or human cancer cell lines [19-23]. This technology has led to the creation of a database of protein antigens that are associated with and specific to a variety of cancers. However, the native immune response to these antigens is not identified or captured by this methodology. Therefore, although proteins that are associated specifically with cancer can be pinpointed, the antibodies that can effectively target these antigens remain mostly unidentified.

To overcome this limitation we designed and implemented an alternate strategy that relies on a unique trioma fusion partner cell line, MFP-2, which we developed [24]. MFP-2 can efficiently fuse with both peripheral blood and lymph node lymphocytes. Following fusion, surviving hybridoma clones are stable for prolonged periods and many produce significant quantities of human monoclonal antibodies. We employed this unique fusion partner cell line to develop a panel of native autologous fully human monoclonal antibodies (fhMAb) that were culled from the natural repertoire arising in breast cancer patients [25]. These fhMAbs reacted specifically with breast cancer cells and malignant tissues. They are useful not only for identification of the target antigens, but also for immunodiagnostic procedures [26] and eventually for immunotherapy of breast cancer, since they can be produced on an industrial scale.

We identified the protein targets of two of the anti-breast cancer autoantibodies that we isolated, and determined that they target the protein GIPC1. Using our fhMAbs that target GIPC1, we studied its expression in human breast tissue and in cultured cells. We determined that this protein is specifically up-regulated in malignant breast epithelial tissue/cells and in breast cancer cell lines and is not detected in normal breast epithelia or in live primary fibroblast cell lines. Therefore, GIPC1 is a novel breast cancer-associated antigen that may play a role in the initiation and/or progression of breast cancer.

## Methods

### Cell culture

All human cancer and normal cell lines were purchased from ATCC. Human breast cancer cell lines MCF-7 and SK-BR-3 and primary human fibroblasts are among those used in this study. SK-BR-3 were cultured in McCoy's 5a medium supplemented with L-glutamine and 10% FBS. MCF-7 was grown in MEM medium supplemented with L-glutamine, non-essential aminoacids, 10% FBS and 0.01 mg/ml bovine insulin. Fibroblasts were cultured in DMEM, supplemented with L-glutamine and 10% FBS. Other normal and neoplastic cell lines were cultured according to the conditions recommended by the ATCC. Hybridoma clones were produced and cultured according to previously described techniques [25].

### Antibody characterization

The isotype of human Abs was determined by ELISA using murine anti-human isotype-specific MAbs to μ-, γ-, κ- and λ-chains (Sigma, USA) and goat anti-mouse Ig (25 μg/mL) conjugated to peroxidase and absorbed with human Ig.

### Immunocytochemistry and immunohistochemistry

Cells were plated on ethanol pre-treated cover slips (Fisher, USA) and placed in 6-well plates (Falcon, USA) in culture medium. After 24 hours the cover slips with attached cells were repeatedly washed in PBS and fixed in ethanol. Following fixation and repeated washes, cover slips were incubated with the primary and secondary antibodies according to standard protocols, stained with propidium iodide 1 μg/ml and analyzed by confocal fluorescent microscopy using a Zeiss Axiovert 100 TV microscope and Zeiss software. For immunohistochemistry randomly selected 5 μm thick sections of paraffin embedded breast cancer tissue were used. Endogenous peroxidase activity was blocked by incubation of slides in 3% H_2_O_2 _in methanol. Following washing, tissue slides were blocked with 5% normal goat serum in PBS. Monovalent Fab fragments of goat anti-human IgM+IgG (Jackson Immunoresearch Laboratories, Inc.), in blocking solution, was then applied for secondary blocking. After 3 washes in PBS, the human monoclonal antibody was applied at an approximate concentration of 5 ug/ml. The slides were then washed and incubated with a second FITC conjugated antibody to human κ-light chains (Sigma, USA) and propidium iodide at 1 μg/ml. Following a few washes, mounting medium (Biomeda, USA) and cover slips were applied and sections were analyzed by standard fluorescent microscopy.

### Western blotting

Cells were lysed with freshly prepared ice cold lysis buffer [20 mM Tris-HCl, pH 7.6, 420 mM NaCl, 0.25% NP40, 2 mM phenylmethylsulfonyl fluoride, 1 ug/ml leupeptin, 250 U/ml Trasylol (aprotinin)] and stored at -80°C or used immediately. Protein concentration was determined with the BioRad Protein Detection Reagent (BioRad). Tissue samples were mechanically homogenized on ice, spun down at 3000 g for 30 min at 4°C and the lower (non lipid) phase was used for further analysis.

Equal amounts of protein were separated on 10% SDS polyacrylamide gels and either Coomassie blue or silver stained according to established techniques [27]. Following electrophoresis, the proteins in the gel were transferred to a nitrocellulose membrane using a variation of the methods of Towbin [28] and Burnette [29], and following blocking, probed with relevant primary and HRP-conjugated secondary antibody. Membranes were processed using an enhanced chemiluminescence kit (ECL, Amersham), and visualized on Kodak BioMax MR-1 film. The immunoblotting with recombinant GIPC1 protein was carried out as previously described [26].

### Binding of ^125^I-labeled monoclonal antibody to SK-BR-3 cells and Scatchard analysis

Antibody 27.F7 was labeled with Na-^125^I (specific activity 17.4 mCi/mg) (New England Nuclear, MA, USA) using Iodogen as previously described [30]. The resulting specific activity of ^125^I-27.F7 was (100 mCi/mmol). SK-BR-3 cells were grown in 24-well plates supplemented with DMEM media with 10% FCS and used in these experiments at subconfluent phase at a density of 2 × 10^5 ^cells per well. Cell monolayers were suspended with trypsin, cooled to 4°C by placing them on ice and washed twice with PBS containing 1% BSA (Sigma, USA). The cells (50,000 cells per sample) were blocked with 1% BSA-PBS for 1 hour at 4°C followed by incubation with ^125^I-27.F7 (approx. 10^5 ^cpm per sample) in the presence of increasing concentrations of cold unlabeled 27.F7 (ranging 0.1 – 200 ng/ml) for 1 hour at 4°C. After incubation the samples were applied to Millipore filters using Millipore 96-well membrane plates (Millipore, USA). The wells were broken off and counted individually in a Cobra γ-counter (Hewlett Packard, USA). Maximum binding, B_max_, was determined by incubating varying numbers of cells (ranging from 1.25 × 10^4 ^to 32 × 10^5 ^cells) with radiolabeled 27.F7 antibody. Nonspecific binding of the tracer was determined in the presence of an excessive amount of unlabeled antibody (500 μg/ml) and was generally less than 5% of maximum binding. All experimental measurements of K_a _and the number of antigen targets per cell were done in triplicate. Analysis of the data was performed according to previously described methods [31-33].

### Antigen identification

RNA was purified from SK-BR-3 cells according to standard protocols. Preparation of mRNA was performed by oligo dT chromatography with a kit from Stratagene (La Jolla, CA). A lambda phage expression library was prepared in lambda ZAP (Stratagene, La Jolla, CA) and plaque lifts were screened with human monoclonal 27.B1 and 27.F7 according to previously described methods [21,34]. Phage plaques that were positive on the first screened were picked and two rounds of plaque purification was performed to ensure that they were true positives. Positive lambda phage clones were autoexcised according to the protocol provided by Stratagene, grown as plasmids according to standard protocols and sequenced to identify the cDNA inserts.

### Northern blot analysis

Total cellular RNA was isolated by the Guanidinium/Phenol extraction method and Northern blotting was performed as previously described [35,36]. Briefly, 15 μg of RNA is denatured and electrophoresed in a 1.2% Agarose gel along with 3.5% formaldehyde, transferred to a nylon membrane and hybridized sequentially with ^32^P-labeled cDNA probes. The GIPC1 cDNA fragment that we isolated using SEREX technology was used for the gene specific probe and a cDNA fragment of the GAPDH gene was used as an internal control to normalize expression. Following hybridization, the filters were washed and exposed for autoradiography.

## Results

### A native fully human autoantibody to breast cancer identifies a cancer-associated antigen that localizes to the cytoplasm and membrane

**Figure 1:**
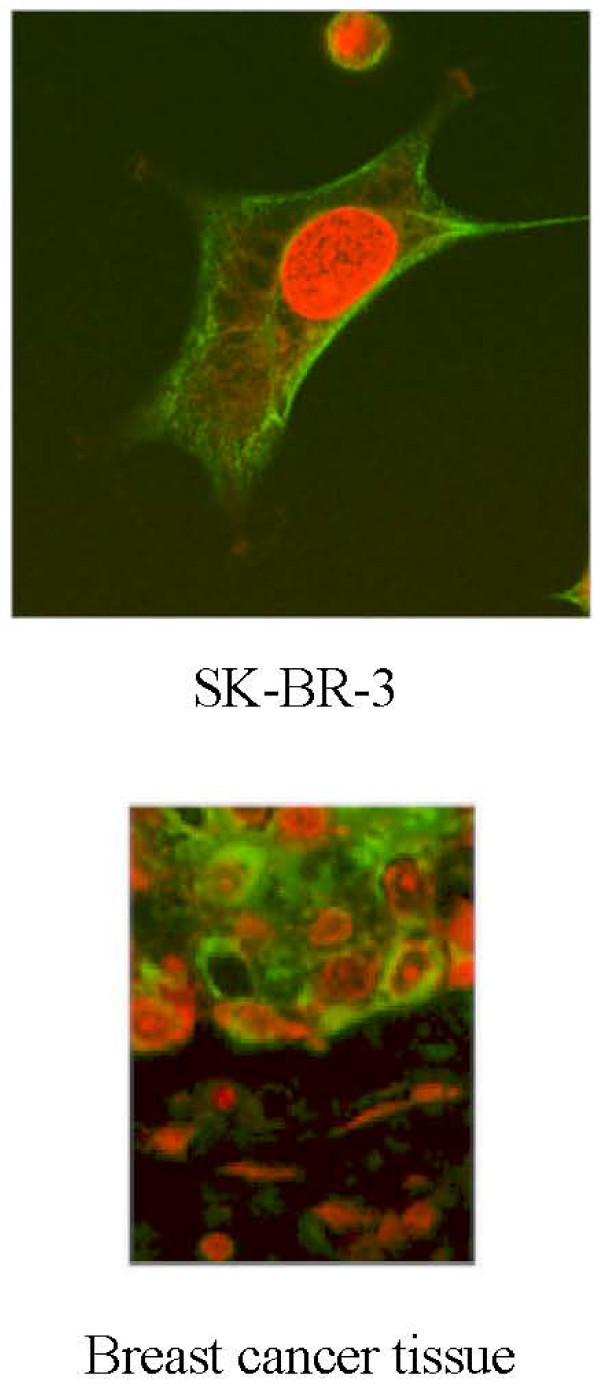
**Human monoclonal 27.B1 stains the membrane and cytosol**. Staining of SK-BR-3 cells and breast cancer tissue was performed with human monoclonal 27.B1. Staining of the SK-BR-3 breast cancer cell line was analyzed by confocal microscopy and indicates that the target antigen is present in the membrane and cytoplasm. Staining of human breast cancer tissue was analyzed by standard fluorescent microscopy.

We previously described the construction of a unique fusion Partner cell line, MFP-2, and its use for the immortalization of both human peripheral blood and lymph node B-lymphocytes [24,25]. MFP-2 was employed for the generation of hybridoma cells from lymphocytes of breast cancer patients that produce autologous anti-breast cancer specific antibodies. The results of that study are described elsewhere [25]. One of these native human monoclonal antibodies, designated 27.B1 (IgM, k), was chosen for further study. It demonstrated an intensely positive reactivity with two human breast cancer cell lines, SK-BR-3 and MCF-7 and no reaction with normal diploid primary human fibroblasts as tested by cELISA (In cELISA whole cells are used in place of a purified antigen as in ELISA) [25]. Confocal microscopy with 27.B1 demonstrated the presence of the target antigen throughout the cytosol and in addition staining of the membrane was especially strong (see Figure 1). Furthermore, 27.B1 stained both primary and metastatic breast cancer with a high specificity and sensitivity [25]. These results along with a more detailed immunocytochemical and immunohistochemical analysis are described elsewhere [25].

### The target antigen for monoclonal antibodies 27.B1 and 27.F7 is a 42 kDa protein

**Figure 2:**
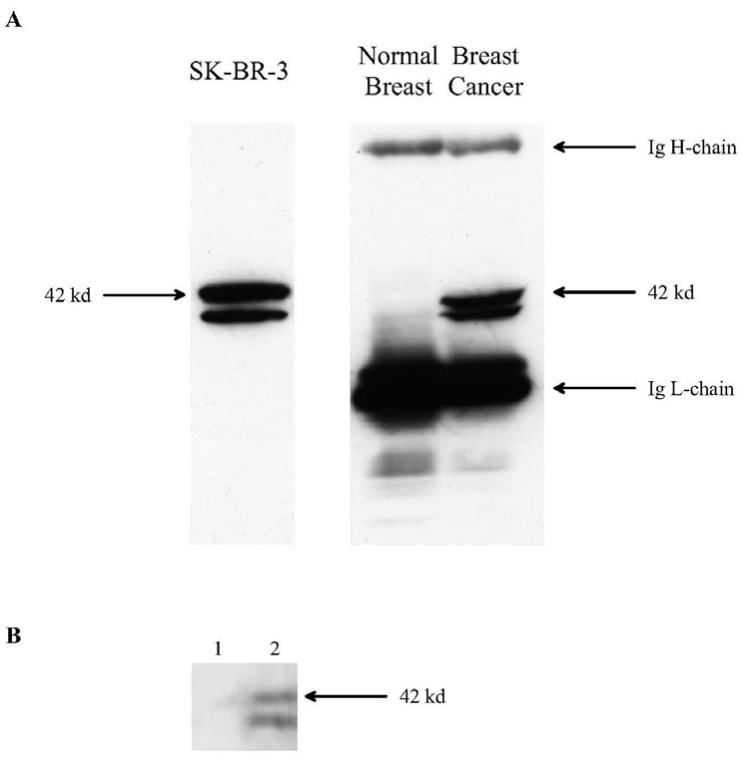
**The target antigen for human monoclonal 27.B1 is a GIPC1 protein**. A. The target antigen for monoclonal 27.B1 in SK-BR-3 cells, breast cancer tissue and normal breast tissue was identified by Western blot and is displayed. Human Ig H and L chains are present in the tissue and are recognized by the secondary anti-human antiserum. The target antigen is detected as a doublet in both the breast cancer tissue and SK-BR-3 cell line but is not detected in normal breast tissue. Both bands in the doublet are present in all breast cancer cell lines analyzed by Western blot but their intensity is variable. B. Immunoblotting with 27.B1 antibody of total cell lysates prepared from SK-BR-3 cells. 27.B1 antibody was preincubated with recombinant GIPC1 protein prior to blotting (lane 1), and compared to non-preincubated control (lane 2).

To identify the size of the 27.B1 target protein, Western blot analysis was performed using whole cell lysates from different cell lines and tissues. Cell lysates prepared from human breast cancer, normal breast tissue, human prostate cancer and two human fibroblast cell lines were run on PAGE under reducing conditions and blotted with fhMAb 27.B1. The antibody reacted with a protein band of approximately 42 kDa molecular weight that is detectable primarily in breast cancer cells (see Figure 2-A). There was no detectable immunoreactivity with the human fibroblasts' lysates and prostate cancer cell line LnCaP whereas only traces of immunoreactivity were detected to prostate cancer cell lines PC3 and DU-145 (data not shown). The protein band revealed by 27.B1 appeared as doublet with a dominant band that migrates slower on a gel. The doublet pattern was not the same in all 27.B1 positive cells; MCF-7 cells displayed the higher molecular weight band in much greater abundance, whereas SK-BR-3 showed both bands in more equivalent intensity (data not shown). Western blot analysis of the same cell lysates under non-reducing conditions displayed no difference in staining pattern, indicating that accessibility of the epitope bound by 27.B1 is disulfide bond independent and likely conformation independent.

Interestingly, we determined that another antibody, 27.F7, which was previously identified as binding breast cancer cells and tissue with high specificity and sensitivity [25], identified the same 42 kDa molecular weight doublet as 27.B1. Furthermore, this antibody detected the bands in the doublet with the same variability in different cell lines as 27.B1. To test the epitope specificity of the two antibodies one of them, 27.F7 was radiolabeled with ^125^I and competitive Western blotting was performed with both antibodies (data not shown). Pretreatment of a SK-BR-3 cell lysate blot with unlabeled 27.F7 inhibited binding of ^125^I-labeled 27.F7, whereas unlabeled 27.B1 antibody did not inhibit binding. This suggests that if these two antibodies are binding the same protein they likely bind different epitopes.

To identify the molecular target(s) for 27.B1 and 27.F7, SEREX technology was applied as previously described [21,25,34] to a cDNA expression library prepared from SK-BR-3 mRNA. Expression clones staining positively with 27.F7 and 27.B1 were selected and the cDNA sequences were found to encode the protein known as GIPC1, following a BLAST algorithm homology search [37]. This protein was previously identified as being involved in the regulation of G protein signaling [38]. The sequence of the cloned cDNA inserts are identical to the respective sequence reported in GenBank for GIPC1.

To confirm that the 42 kDa band demonstrated in Figure 2-A is indeed GIPC-1, we performed immunoblotting with recombinant GIPC-1 protein (Figure 2-B). For this purpose, 27.B1 antibody was preincubated with a bacterial expressed and refolded recombinant GIPC1 protein prior to blotting (Figure 2-B, lane 1) and compared to non-preincubated control (Figure 2-B, lane 2). These results demonstrate that fhMAb 27.B1 binding to the same 42 kDa band was specifically inhibited by the recombinant protein and this confirmed the specificity of this antibody to the GIPC1 antigen.

### Scatchard analysis

**Figure 3:**
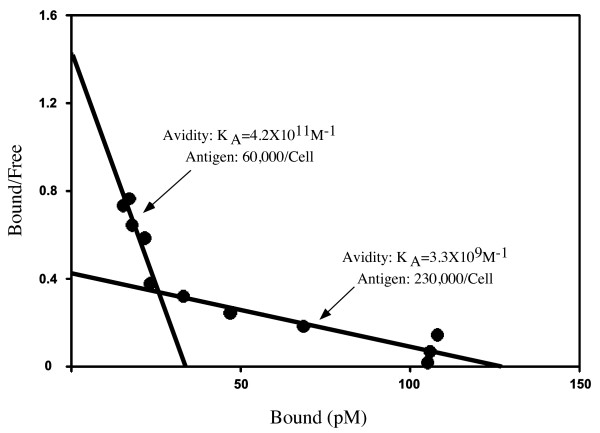
**Scatchard analysis of human anti-GIPC1 monoclonal antibody on antibody on SK-BR-3 cells**. Scatchard analysis of human monoclonal antibody 27.F7 performed on SK-BR-3 cells revealed the presence of an antigen target with two affinities. This suggests that two populations of GIPC1 molecules exist in these cells and may be related to the protein doublet identified by Western blot analysis in Figure 2.

Scatchard analysis of ^125^I-27.F7 binding to SK-BR-3 cells revealed a two-mode pattern of binding, which matches a model with binders of two different avidities (see Figure 3) [31]. One type of bound ligand is represented by approximately 20% of all 27.F7 targets and binds the antibody with high avidity (K_a _= 4.2 × 10^11 ^M^-1^). The second type of ligand binds with a lower avidity (K_a _= 3.3 × 10^9 ^M^-1^) and constitutes about 80% of the total antigen molecules. An estimate for the total number of antigen molecules per cell is approximately 3 × 10^5 ^target GIPC1 molecules per cell. The identification of two binding avidities may be related to the fact that human monoclonal antibody 27.F7 (and 27.B1) identifies a two band doublet on a Western blot (see Figure 2). This suggests that one of the bands may be a ligand of higher avidity while the second one is of lower avidity. The doublet itself has been previously reported although the reason for two bands has not been determined [38]. It might be explained by an alternative start codon or posttranslational modification.

### GIPC1 is up-regulated in breast cancer cell lines

**Figure 4:**
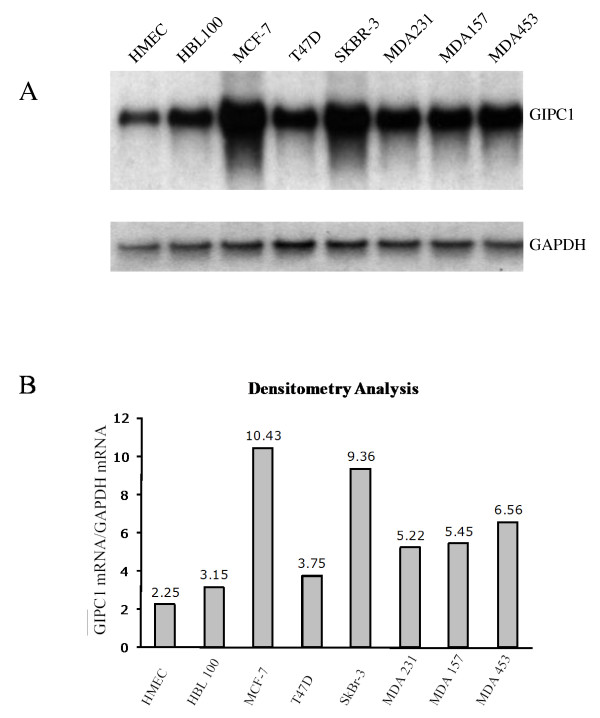
**GIPC1 RNA expression analysis in normal and neoplastic cell lines**. Panel A: Northern blot analysis of total RNA was performed with RNA samples from a human microvascular endothelial cell line (HMEC), normal breast epithelium cell line HBL100 and breast cancer cell lines MCF-7, T47D, SK-BR-3, MDA231, MDA157 and MDA453. A probe for the GAPDH gene was used to normalize expression. Panel B: Densitometry analysis of the Northern blot was performed to quantitate the mRNA expression. The data indicates that the GIPC1 gene is upregulated in breast cancer cell lines.

The strong staining by the human anti-GIPC1 monoclonal antibodies, 27.B1 and 27.F7, on breast cancer cell lines and absent staining of normal cells [25] suggests that the protein might be up-regulated. To semi-quantitatively examine gene expression we performed Northern blot analysis to determine if a higher level of GIPC1 specific mRNA was indeed present in the breast cancer cell lines relative to normal cell lines. RNA from a variety of breast cancer cell lines along with non-neoplastic cell lines was blotted and GAPDH expression was monitored as an internal control to normalize expression. The results are depicted in Figure 4. They indicate that breast cancer cells indeed have increased expression of GIPC1 specific RNA relative to that of normal cell lines.

## Discussion and conclusion

Our studies have determined that GIPC1 is a novel breast cancer associated antigen that is up-regulated in breast cancer cell lines and in malignant tissue from all breast cancer patients tested in this study. In our previous studies [25], we determined that the reactivity of the human monoclonal antibodies 27.B1 and 27.F7 are specific to breast cancer tissue and cells. Our present study demonstrated that these antibodies identify the unique antigen target GIPC1 (GAIP interacting protein, C domain), a PDZ domain containing protein [38,39].

GIPC1 was first described as a PDZ domain protein that binds to the C terminus of RGS-GAIP (for regulators of G signaling – Gα_i3 _interacting protein) [38]. GAIP itself is considered to be a Gα_i3 _regulator, which acts as GTP-ase activating protein switching Gα_i3 _into an inactive mode [40-45]. Although the functional pathway of Gα_i3 _is apparently vesicular trafficking, with GAIP serving as its regulator, the physiological relevance of the interaction between GIPC1 and GAIP is been actively investigated [46-53].

With respect to breast tissue, the recognition by 27.B1 and 27.F7 of GIPC1 is strictly limited to neoplastic cells. Furthermore, the subcellular localization of the bound antibodies in the cell membrane and cytoplasm is consistent with what has been previously described for GIPC1 localization [46]. Taken together, our findings suggest that the up-regulation of GIPC1 is cancer cell specific. Although 27.B1 does not detect any protein by FACS or Western blot analysis in normal cells and tissues it is likely below the level of detection since GIPC1 is likely involved in many diverse cellular processes [47,49-55].

GIPC1 plays a role in mediating the assemblage of molecules involved in signaling transduction pathways [53]. As such, these molecules are involved in protein-protein interactions and likely modulate the activity of their targets [53]. Proteins containing PDZ domains, and the interactions that they mediate, may be involved in a wide variety of signal transduction cascades including interaction with receptors, adhesion molecules, ion channels, gap junctions, cytoskeleton proteins and other vital proteins, such as Fas [48-54,56]. Moreover, GIPC1 appears to be a highly conserved protein. In rodents it regulates distribution of M-Sem-F, a neuronal membrane-associated protein and binds to a glucose transporter protein, GLUT1 [57,58]. It is tempting to speculate on the role of over-expressed GIPC1 in binding to a glucose transporter protein with the subsequent influx of glucose supporting growth of tumor cells. Of course, other proteins may be linked to GIPC1 function in cancer cells; this is currently under investigation in our laboratory.

Research of cancer-associated antigens is an extremely important pursuit. Identification of these antigens can provide insight into the cause of a malignancy, identify targets for immunotherapy and immunodiagnostics [26] as well as lead to the development of new cancer vaccines. Previous studies on natural monoclonal autoantibodies from cancer patients did not describe the target antigens [59,60]. Our studies demonstrate that by combining the "immunoprospecting" of cancer autoantibodies and SEREX technology discovery of target antigens for monoclonal cancer autoantibodies can be accomplished. In conclusion, our studies revealed that GIPC1 is a novel cancer-associated antigen; its role in carcinogenesis, however, needs further clarification. It also needs to be clarified whether GIPC1 is a specific breast cancer-associated antigen or it is overexpressed in other malignant diseases as well.

## Competing interests

The authors declare that they have no competing interests.

## Authors' contributions

SR carried immunochemical and biochemical studies and organized all the data for the manuscript; MS carried the molecular biology experiments and performed SEREX for identification of the antigen; GK developed hybridoma clones producing specific monoclonal antibody; VY performed flow cytometry and Western blot studies for confirmation of the antigen identity; CL did cell culture work related to cloning and selection of antibody-producing clones; AE, LO and GLC provided an expert clinical information on breast cancer and contributed to the interpretation of data and consideration of potential applications; LL and IT are senior co-investigators who conceived the study, developed its design participating in its coordination and drafting the manuscript. All authors read and approved the final manuscript.

## Pre-publication history

The pre-publication history for this paper can be accessed here:


